# *In vitro* Differentiation of Hair Follicle Stem Cell into Keratinocyte by Simvastatin

**DOI:** 10.29252/ibj.23.6.404

**Published:** 2019-11

**Authors:** Azar Babakhani, Paria Hashemi, Javad Mohajer Ansari, Parisa Ramhormozi, Maliheh Nobakht

**Affiliations:** 1 Department of Anatomy, Faculty of Medicine, Iran University of Medical Sciences, Tehran, Iran;; 2 Antimicrobial Resistance Research Center, Institute of Immunology and Infectious Disease, Iran University of Medical Sciences, Tehran, Iran

**Keywords:** Cell differentiation, Keratinocytes, Simvastatin

## Abstract

**Background:**

Hair follicle stem cells (HFSCs) located in the bulge area has shown to be highly proliferative and could differentiate into neurons, glia, smooth muscle cell, and melanocytes *in vitro*. Simvastatin is an HMG-CoA reductase inhibitor that exerts pleiotropic effects beyond simple low-density lipoprotein lowering and has a similar impact on the differentiation of bone marrow stromal cells and peripheral blood mononuclear cells. The present study examined the hypothesis that the application of simvastatin would induce the HFSCs differentiation into keratinocyte.

**Methods:**

The bulge of the hair follicle was anatomized, and HFSCs were cultivated. The flow cytometry and immunocytochemical staining for detection of nestin, CD34, and Kr15 biomarkers were performed before differentiation. In order to hasten the HFSCs differentiation to keratinocyte, HFSCs were treated with 1 µM, 2 µM, and 5 µM of simvastatin daily for a week. After differentiation, the flow cytometry and immunocytochemical staining were performed with Kr15 and Kr10 biomarkers, and the MTT assay was carried out as an index of cell viability and cell growth.

**Results:**

Our results showed that bulge of HFSCs were nestin and CD34 positive and Kr15 negative. Simvastatin significantly increased the viability of HFSCs (*p* < 0.05) at the concentration of 5 µM. In addition, the percentages of keratinocyte-differentiated cells treated with 5 µM of simvastatin showed a significant increase compared to all other treated groups (*p* < 0.05).

**Conclusion:**

Our findings demonstrate that 5 µM of simvastatin could induce HFSCs differentiation into keratinocyte.

## INTRODUCTION

Wound healing is one of the major therapeutic and economic issues in the field of regenerative medicine^[^^1^^]^. Epithelialization is a critical parameter in wound healing^[^^2^^,^^3^^]^. Keratinocytes, as a major cellular constituent of the epidermis, are responsible for repairing the injured epidermis^[^^4^^]^. Differentiation of hair follicle stem cells (HFSCs) into keratinocytes highlights a promising stem cell-based therapy in the field of wound healing. Adult HFSCs are more accessible, because they are not tumorigenic and have the immunomodulatory capacity^[^^5^^,^^6^^]^. HFSCs were found to contribute to wound healing after skin injury by migration and differentiation into epidermal cells^[^^7^^]^. Studies have shown strong multipotency of bulge stem cells, where they were attracted to differentiate into neurons, glial cells, keratinocytes, melanocytes, and mesenchymal cells; also, they were detected to contribute to angiogenesis^[^^8^^-^^12^^]^. 

Statins, 3-hydroxy-3-methylglutary1-coenzyme A (HMG-CoA) reductase inhibitors including simvastatin, are among the most prescribed drugs in the world^[^^13^^]^. Reducing the synthesis of mevalonate and isoprenoid, due to the inhibition activity of HMG-CoA reductase, is leading to the reduction of cholesterol production, and it is responsible for pleiotropic effects of all statins^[^^14^^]^. Simvastatin applies pleiotropic effects on treatment of high plasma cholesterol levels^[^^14^^,^^15^^]^. The influences of statins on cell differentiation have been mentioned in some studies; the results showed that simvastatin promotes osteoblast viability and adipocyte differentiation^[^^16^^]^ and enhances endothelial differentiation of peripheral blood mononuclear cells^[^^17^^]^. In addition, simvastatin has useful impacts on curing wound that include anti-inflammatory, anti-bacterial, immunomodulatory, anti-oxidative effects, as well as the improvements of microvascular and reperfusion function^[^^1^^,^^2^^]^. In this work, for the first time, we assessed the effects of simvastatin on HFSCs differentiation into keratinocyte by using immunocytochemical and flowcytometric analyses.

## MATERIALS AND METHODS

**Animals**

All animal handling procedures were performed based on the guidelines of the Iranian Council for Use and Care of Animals, and they were approved by the Ethical Committee of Iran University of Medical Sciences, Tehran, Iran (grant no. 28412). The animals were allowed to have free access to food and water at all times and were kept under 12-hour light/dark cycles during the experimentations.

**Cell culture**

Hair follicles were collected from whiskers of male Wistar rats (n = 10, body weight = 200-250 g) and digested with collagenase I/dispase II solution (Sigma-Aldrich, Germany) based on a previously described method^18]^. The bulge of the hair follicle was dissected under a microscope by two transverse cuts, which were made above and below the follicle, and then was cultured in Dulbecco’s modified Eagle’s medium/F12 supplemented with 10% fetal bovine serum (FBS), 10 ng/ml of epidermal growth factor (Sigma-Aldrich), 100 µg/ml of penicillin-streptomycin (Gibco, Carlsbad, CA, USA), 0.5 mg/ml of hydrocortisone, and 0.1 U/ml of insulin. The bulges were allowed to attach to the collagen type I-coated tissue culture flasks. For six days after expatriation, bulge fragments were removed, and adherent cells were incubated (5% CO_2_, 37 °C, three days) in the same medium. 

**MTT assay**

In this study, different concentrations of simvastatin (0-30 µM) were used to evaluate its cytotoxicity on HFSCs and to find the suitable concentration for keratinocyte induction. For this, HFSCs (5000 cell/well) were cultured in a 96-well plate overnight, and 0.5-30 µM of simvastatin was added to each culture in a triplicate sample form. Untreated cells were used as controls. After 18 h of culture, 10 µL of MTT (5 mg/ml, Sigma Aldrich) was added to each well, followed by incubation at 37 °C for 4 h. The MTT solution was replaced with 150 µL of DMSO at 37 °C for 5 min. Finally, the survival portion of HFSCs was measured at 570 nm using an ELISA reader.

**Incubation with simvastatin**

After six days of culture, HFSCs were incubated in a fresh medium containing 5 µM of simvastatin (Biocon Limited, India), dissolved in ethanol plus 10 mg/ml of epidermal growth factor without FBS and treated for a week. The medium was changed daily during the differentiation period. Cell differentiation was assessed by counting cells using immuno-cytochemistry technique at 14 days after culture.

**Immunocytochemical staining**

Cell cultures were fixed in 4% paraformaldehyde at room temperature for 10 min. After washing three times with PBS, the cells were incubated in a blocking buffer (10% goat serum, Sigma-Aldrich; 0.3% Triton X-100, Fluka, USA) at room temperature for 1 h. Then the cells were incubated at 4 °C overnight with the following primary antibodies: rabbit anti-nestin monoclonal antibody (1:200), rabbit anti-CD34 antibody (1:75), rabbit anti-Kr15 antibody (1:75), and rabbit anti-Kr10 antibody (1:100), all from Sigma Aldrich. Fluorescein isothiocyanate-conjugated secondary goat anti-rabbit antibody (1:1400, Abcam, Cambridge, UK) was added to the cells in the dark at 37 °C for 1 h. As cell nuclei counterstained, the cells were stained with 10 µg/ml of propidium iodide (Sigma) in the dark for 1 min. After washing, labeled cells were observed with fluorescent microscopy (Olympus Ax 70, Japan).

**Flow cytometry assay**

Flow cytometry method was used for confirming the results of the immunocytochemical assay. After detaching from the bottom of the flasks, the cells were centrifuged and incubated in the same primary antibodies at room temperature for 1 h. Subsequently, the fluorescein isothiocyanate-conjugated IgG (1:1400, Abcam) was added to the cellular plaque tube, and the final mixture was incubated in the dark at 37 °C for 1 h, and the suspension was poured in the flow cytometry tubes. The negative control samples cytometry tubes. The negative control samples were prepared using cells that were not incubated with primary antibodies.

**Fig. 1:**
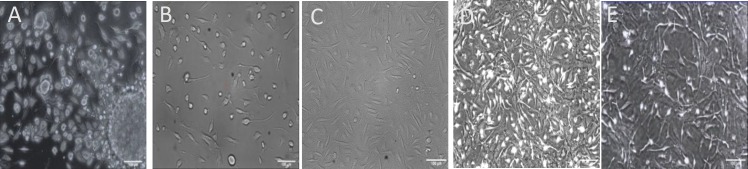
The primary culture of bulge hair follicle stem cells (HFSCs) from rat hair follicle. (A) HFSCs 3-4 days after the primary culture; (B and C) migration and proliferation of HFSCs after the colony formation; (D and E) HFSCs culture after 9 and 10 days (Scale bar A and B = 20 µm; C, D, and E = 100 µm)

**Statistical analysis**

Results were presented as a mean ± standard deviation. Data analyses were performed using one-way ANOVA, followed by Tukey's post-hoc. Statistical analysis was carried out using Prism 6 for windows. For all tests, a *p* value of less than 0.05 was considered statistically significant.

## RESULTS

**Characterization of cultures of rat HFSCs**

**Fig. 2:**
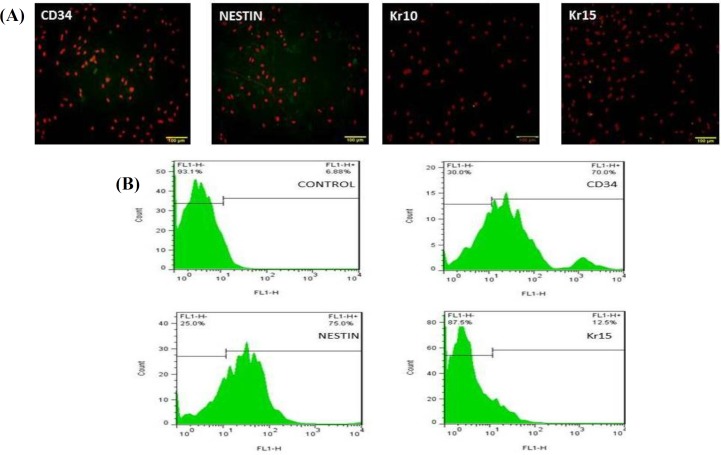
Analyzing bulge cells before differentiation. (A) Hair follicle stem cells (HFSCs) showed positive reaction in immunocytochemistry staining with CD34 and nestin antibodies, but no positive reaction was observed in immunocytochemistry staining with Kr15 and Kr10 antibodies (scale bars = 100 µm; (B) flow cytometry assay from the surface adhesion molecules on HFSCs with nestin, CD34, and Kr15 antibodies before differentiation. Flow cytometry results showed the percentage of undifferentiated cells to be 70% and 75% for expression of CD34 and nestin, respectively and 12.5% for expression for Kr15. Incubated cells with only secondary antibody have been considered as negative controls

Rat HFSCs were isolated from whisker follicle and cultivated successfully. As shown in Figure 1A on the third day of hair follicle bulge culture, the stem cell started to outgrowth, and numerous cells were migrated from the hair follicle bulge. The cells showed cobblestone or round morphology, which is typical of epithelial cells (Fig. 1A). After 4-6 days of culture, the cells were spindle-shaped and firmly adhered to the flasks (Fig. 1B). On day 6 of culture, the cell number increased and formed a homogeneous population from the bulge HFSCs, at the bottom of cell culture flasks (Fig. 1C-E). These findings suggested that after 6-9 days of the primary culture (three passages), HFSCs had the morphology and characterization of the stem cells. Two methods, namely immuno-cytochemistry and flow cytometry, were used to confirm these cells were primitive stem cells. The results showed that bulge cells were CD34 and nestin-positive, but Kr15 and Kr10 were negative (Fig. 2).

**Promotion of HSFSCs survival**

**Fig. 3:**
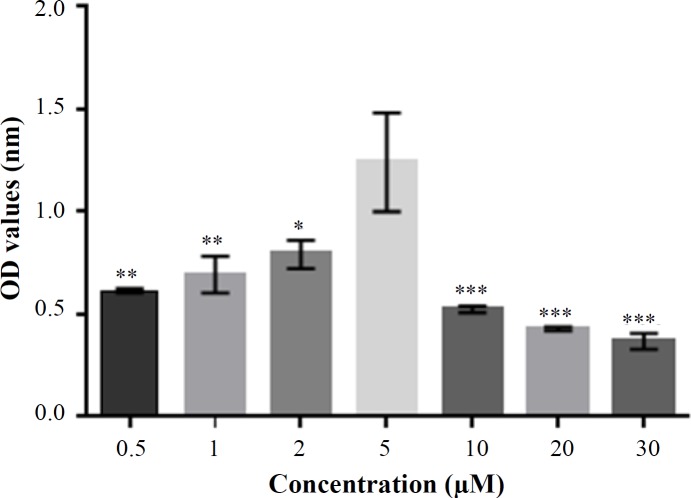
MTT assay results. A significant difference was observed between the group treated with 5 µM of simvastatin and other groups. These results suggested that 5 µM concentration of simvastatin promoted the HFSCs proliferation and had no cytotoxic effects (one-way ANOVA, Tukey's test; *p* < 0.01 [5 µM vs. 0.5 µM and 1 µM], *p* < 0.05 [5 µM vs. 2 µM], *p* < 0.001 [5 µM vs. 10, 20, and 30 µM]). Error bar represents mean ± SD; ^*^p < 0.05 (5 µM vs. 2 µM);^**^P < 0.01 (5 µM vs. 0.5 and 1 µM); ^***^p < 0.001 (5 µM vs. 10, 20, and 30 µM)

MTT chromometry assay was used to determine the cell viability and to select the most efficient concentration of inducer. Simvastatin was used in a range of 0-30 µM. Results showed that the simvastatin concentrations of 10-30 µM were toxic and inhibited cell growth, but cell viability was higher at concentrations less than 10 µM. However, simvastatin at concentration of 5 µM had the highest optical density and showed a significant increase in the viability of HFSCs compared to all other groups (one-way ANOVA and Tukey’s test, *p* < 0.001; Fig. 3)

**Quantitative analysis of differentiated cells**

**Fig. 4:**
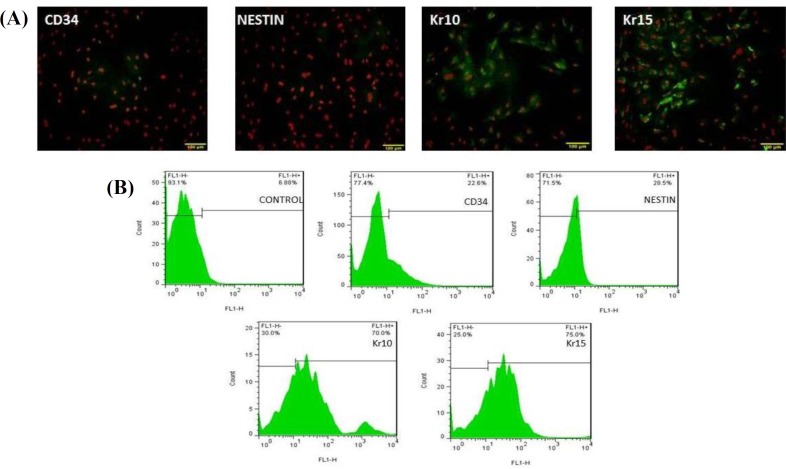
Analysis of differentiated cells after induction with 1 µM concentration of simvastatin. (A) Treated cells show low levels of nestin and CD34 immunoreactivity, but Kr10 and Kr15 were expressed in treated groups (scale bars = 100 µm); (B) flow cytometry results after induction of 1 µM concentration of simvastatin. Flow cytometry results showed the percentage of differentiated cells to be 75% and 70% for the expression of Kr15 and Kr10 in induced group and to be 28.5% and 22.6% for the expression of nestin and CD34 in treated groups, respectively

**Fig. 5:**
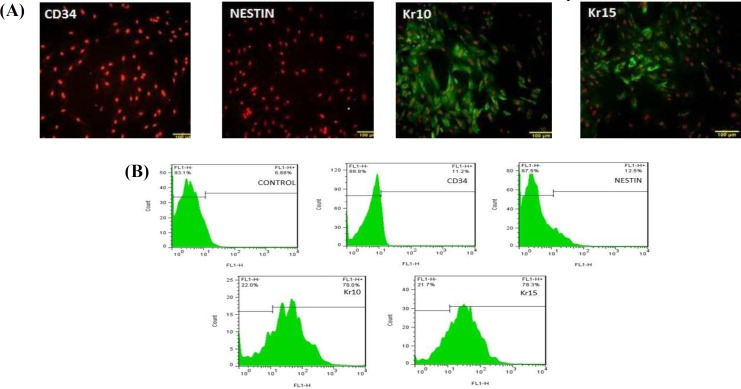
Analysis of differentiated cells after induction with 2 µM concentration of simvastatin. (A) Differentiated cells showed positive reaction in immunocytochemistry staining with Kr10 and Kr15 antibodies, whereas no positive reaction was observed in immunocytochemistry staining with CD34 and nestin antibodies (scale bars = 100 µm); (B) flow cytometry results after induction of 2 µM concentration of simvastatin. Flow cytometry results showed the percentage of differentiated cells to be 78.3% and 78% for the expression of Kr15 and Kr10 in induced group and to be 12.5% and 11.2% for the expression of nestin and CD34 in treated group, respectively

**Fig. 6:**
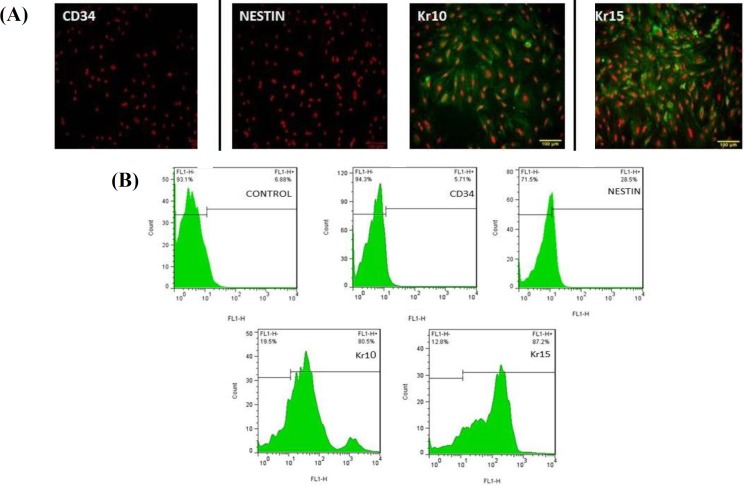
Analysis of differentiated cells after induction with 5 µM concentration of simvastatin. (A) Differentiated cells showed the most expression of Kr10 and Kr15 due to the lack of nestin and CD34-immunoflurescent (scale bars = 100 µm); (B) flow cytometry results after induction of 5 µM concentration of simvastatin. Flow cytometry results showed the percentage of differentiated cells to be 87.2% and 80.5% for the expression of Kr15 and Kr10 in induced group and to be 3.95% and 5.71% for the expression of nestin and CD34 in treated group, respectively

**Fig. 7:**
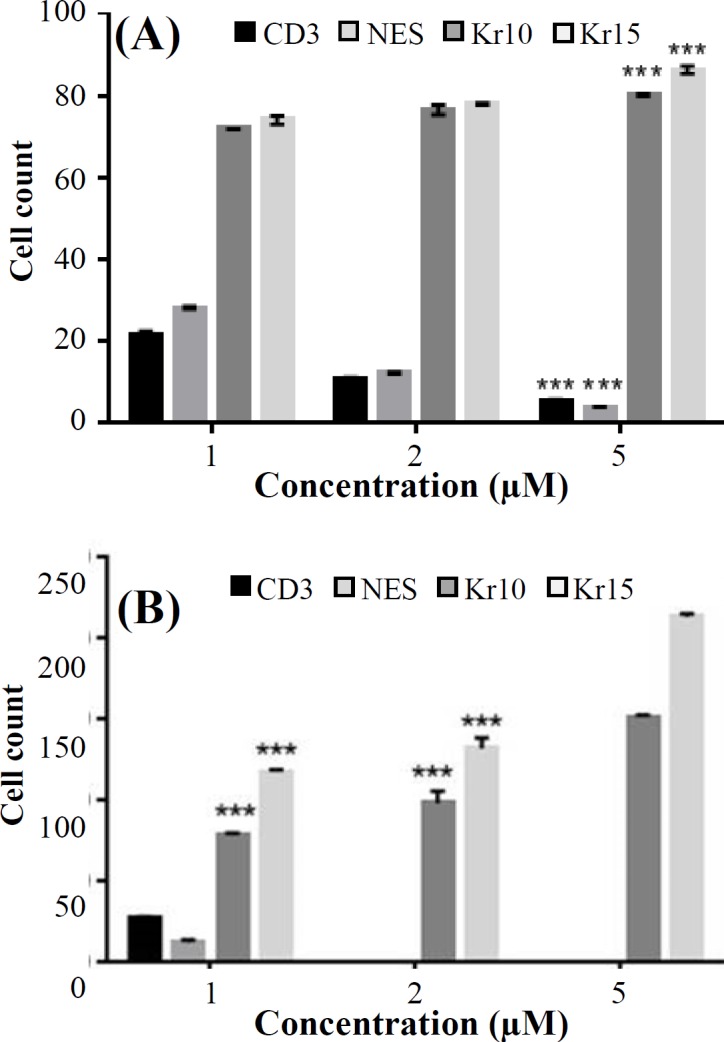
Immunocytochemistry (A) and flow cytometry (B) analyses after the induction of 1 µM, 2 µM, and 5 µM concentrations of simvastatin 14 days after co-culture. The average percentages of differentiated cells were evaluated with Kr15, Kr10 (keratinocyte cell markers) nestin, and CD34 (hair follicle stem cell markers) antibodies. Asterisks indicate that the percentage of the keratinocyte differentiating from the hair follicle stem cells in 5 µM of does-treated group was more than other groups (two-way ANOVA, Tukey’s test; ^***^p < 0.001; 5 µM vs. 1 µM and 2 µM). Error bars represent mean ± SD

For measuring the number of cells differentiating from HFSCs, immunocytochemical staining was performed using Kr15 and Kr10 (specific markers of keratinocyte cells) and nestin and CD34 (specific markers of HFSCs). On day 6 after cultivation, treated groups were fed with three different doses of simvastatin every day for 1 week. Immuno-cytochemical results on the 14^th^ day after cultivation showed that HFSCs differentiated into keratinocyte (Figs. 4, 5, and 6). Kr10 and Kr15 were expressed in treated groups. Differentiated rat HFSCs showed the low levels or lack of nestin and CD34 immuno-reactivity. However, in the concentration of 5 µM, simvastatin significantly increased the rate of HFSC differentiation at day 14 after culture (*p* < 0.001; Figs. 7A). Using the concentration of 5 µM of simvastatin, most of the cells including keratinocyte cells in aggregates had the most expression of Kr15 (mean 213.50 ± 1.87) and Kr10 (mean151.83 ± 1.16), while no nestin and CD34 immunofluorescent was detected (Fig. 6). At the lower concentration, simvastatin (1 µM and 2 µM) also induced an increase in the number of differentiated cells on day 14 after culture, which was not statistically significant (Figs. 4 and 5). Flow cytometry analysis confirmed these results and showed a significant increase in the percentage of differentiated cells treated with 5 µM of simvastatin compared with the other groups. Figure 7B shows the average percentage of differentiated cells, which were evaluated with Kr15 and Kr10 (as keratinocyte cell markers) and nestin and CD34 (as HFSC markers) antibodies.

## DISCUSSION

The major challenges in the field of tissue engineering are epithelial replacement and epithelialization, especially in serious skin injuries resulting from the diabetic ulcer or burns^[^^19^^]^. The use of stem cells to enhance tissue and organ regeneration is an important factor in tissue engineering^[^^12^^,^^20^^]^. HFSCs are very appropriate stem cells due to some factors such as multipotency and high proliferative potential, as well as ability to differentiate into neurons^[^^10^^]^, keratinocytes^[^^21^^,^^22^^]^, and endothelial cells^[^^23^^]^. Additionally, because many clinical applications of HFSCs are for the enhancement of wound healing^[^^8^^]^, promotion of nerve repair, and functional recovery of injured peripheral nerve and spinal cord^[^^24^^]^, it may even be the stem cell of choice in regenerative medicine in the future. All of these benefits in addition to easy culture and expansion, rapid availability, and high proliferative potential of HFSCs^[^^18^^]^ eventually led us to select this source of stem cell in the present study. Since keratinocytes play an important role in wound healing, committing stem cells to the keratinocyte lineage could be feasible by various strategies, such as inducing of exogenous cytokines, growth factors, and chemicals. 

Simvastatin is an HMG-COA reductase inhibitor and exerts various biological functions ranging from simple low-density lipoprotein lowering effects to angiogenesis and anti-apoptotic activities^[^^25^^,^^26^^]^. There are few studies reporting the effects of statins on cell differentiation, and to the best of the authors' knowledge, this is the first work showing differentiation activity of simvastatin on HFSCs. In the present study, HFSCs were isolated and cultured successfully from the bulge of rat whisker follicle. The results of flow cytometry and immunocytochemistry staining before differentiation indicated that the cells strongly expressed specific stem cell markers, CD34, and nestin, but they were negative for Kr15, suggesting that these cells are in a relatively undifferentiated state. These results were in agreement with a number of previous studies^[^^5^^,^^8^^,^^12^^,^^18^^,^^22^^]^. After inducing bulge HFSCs with three different concentrations of simvastatin (1, 2, and 5 µM) on day 14 post-cultivation, immuno-cytochemistry staining and flow cytometry assay were performed for identifying the expression of Kr15, as a specific marker of keratinocyte, and Kr10, as a keratin normally expressed in terminally differentiating epidermal keratinocyte^[^^27^^,^^28^^]^, as well as for nestin and CD34, as stem cell markers, in hair follicle bulge.

 Our results showed that Kr15 and Kr10 were highly expressed in all treated cells compared with untreated cells (Figs. 4, 5, and 6). However, the level of expression significantly increased when these cells were induced with 5 µM of simvastatin (*p* < 0.001). Meanwhile, the differentiation induction with 5 µM, 2 µM, and 1 µM of simvastatin indicated the lack or low levels of nestin and CD34-expression as specific stem cell markers. These observations showed that treatment with simvastatin could mediate the keratinocyte differentiation of undifferentiated bulge HFSC *in vitro*.

Independent of low-density lipoprotein cholesterol reduction, statins have several physiological effects. They have the ability to directly alter cellular events rather than cholesterol synthesis^[17,25]^. Previous studies have shown that three major MAP kinase pathways are involved in the process of keratinocytes differentiation, which is activated by multiple stimuli, including calcium influx, tumor necrosis factor, and epidermal growth factor^[^^4^^]^. The increased intracellular calcium leads to enhance transglutaminase activation and cornified envelope formation, the outermost layer of the epidermis^[^^1^^,^^3^^,^^4^^]^. In addition, intracellular calcium ions are accountable for signal transduction pathways, including cell proliferation and cell death^[^^13^^,^^14^^]^. Several investigations have reported that simvastatin causes an increase in cytoplasmic calcium level [Ca2+] i, which is a key factor in cellular processes and mechanisms of keratinocytes differentiation during re-epithelialization^[^^4^^,^^13^^,^^14^^,^^29^^,^^30^^]^. One of the pleiotropic effect of simvastatin is stimulating differentiation activity in different sources of stem cells such as dental pulp stem cells^[^^15^^]^, bone marrow stromal cells^[^^31^^]^, peripheral blood mononuclear cells^[^^17^^]^, and murine embryonic stem cells^[^^32^^]^. Perhaps 5 µM concentration of simvastatin promoted HFSC differentiation into keratinocyte. Since the toxic effect of simvastatin on HFSCs has not been reported yet, we also tested simvastatin cytotoxicity in different doses using the MTT test. MTT is a quantitative assay for cell viability *in vitro* to test the cytotoxicity of biomaterials and different doses of chemicals and growth factors^[^^19^^]^. As shown in Figure 3, MTT analysis indicated that simvastatin resulted in HFSCs death at the concentration ranging from 10 to 30 µM. Besides, 5 µM of simvastatin was found as an optimal concentration for the survival and viability of HFSCs and resulted in significant improvement of cell viability in comparison to all other groups (*p* < 0.001). These findings showed that simvastatin at concentration of 5 µM (in ethanol) had no cytotoxicity and was suitable for HFSCs culture. According to these results, simvastatin (5 µM) can promote keratinocyte viability and differentiation *in vitro* and may be a promising drug for regenerating medicine in the future.
